# Do Patients with Atrial Fibrillation and a History of Ischemic Stroke Overuse Reduced Doses of NOACs?—Results of the Polish Atrial Fibrillation (POL-AF) Registry

**DOI:** 10.3390/ijerph191911939

**Published:** 2022-09-21

**Authors:** Anna Szyszkowska, Łukasz Kuźma, Beata Wożakowska-Kapłon, Iwona Gorczyca-Głowacka, Olga Jelonek, Beata Uziębło-Życzkowska, Paweł Krzesiński, Maciej Wójcik, Robert Błaszczyk, Monika Gawałko, Agnieszka Kapłon-Cieślicka, Tomasz Tokarek, Renata Rajtar-Salwa, Jacek Bil, Michał Wojewódzki, Anna Szpotowicz, Małgorzata Krzciuk, Janusz Bednarski, Elwira Bakuła, Marcin Wełnicki, Artur Mamcarz, Anna Tomaszuk-Kazberuk

**Affiliations:** 1 Department of Cardiology, Medical University of Bialystok, 15-276 Bialystok, Poland; 2 Department of Invasive Cardiology, Medical University of Bialystok, 15-276 Bialystok, Poland; 3 1st Clinic of Cardiology and Electrotherapy, Swietokrzyskie Cardiology Centre, 25-736 Kielce, Poland; 4 Collegium Medicum, The Jan Kochanowski University, 25-369 Kielce, Poland; 5 Department of Cardiology and Internal Diseases, Military Institute of Medicine, 04-141 Warsaw, Poland; 6 Department of Cardiology, Medical University of Lublin, 20-059 Lublin, Poland; 7 1st Department of Cardiology, Medical University of Warsaw, 02-097 Warsaw, Poland; 8 Department of Cardiology, Maastricht University Medical Centre, Cardiovascular Research Institute Maastricht, 6229 ER Maastricht, The Netherlands; 9 Institute of Pharmacology, West German Heart and Vascular Centre, University Duisburg-Essen, 45147 Essen, Germany; 10 Center for Invasive Cardiology, Electrotherapy and Angiology, 38-400 Nowy Sacz, Poland; 11 Cardiology and Cardiovascular Interventions Clinical Department, The University Hospital, 30-688 Krakow, Poland; 12 Department of Invasive Cardiology, Center of Postgraduate Medical Education, 02-776 Warsaw, Poland; 13 Department of Cardiology, Regional Hospital, 27-400 Ostrowiec Swietokrzyski, Poland; 14 Department of Cardiology, St. John Paul’s II Western Hospital, 05-825 Grodzisk Mazowiecki, Poland; 15 3rd Department of Internal Diseases and Cardiology, Medical University of Warsaw, 02-091 Warsaw, Poland

**Keywords:** atrial fibrillation, ischemic stroke, anticoagulation, reduced dose

## Abstract

Background: The aim of our study was to assess if patients with AF (atrial fibrillation) and a history of ischemic stroke (IS) excessively receive reduced doses of NOACs (non-vitamin K antagonist oral anticoagulants). Methods: The Polish AF (POL-AF) registry is a prospective, observational, multicenter study, including patients with AF from 10 cardiology hospital centers. In this study we focused on patients with IS in their past. Results: Among 3999 patients enrolled in the POL-AF registry, 479 (12%) had a previous history of IS. Compared to patients without IS history, post-stroke subjects had a higher CHA_2_DS_2_-VASc score (median score 7 vs. 4, *p* < 0.05). Of these subjects, 439 (92%) had anticoagulation therapy, 83 (18.9%) were treated with a vitamin K antagonist (VKA), 135 (30.8%) with rivaroxaban, 112 (25.5%) with dabigatran, and 109 (24.8%) with apixaban. There were a significant number of patients after IS with reduced doses of NOACs (48.9% for rivaroxaban, 45.5% for dabigatran, and 36.7% for apixaban). In many cases, patients were prescribed reduced doses of NOACs without any indication for reduction (28.8% of rivaroxaban use, 56.9% of dabigatran use, and 60.0% of apixaban use—out of reduced dosage groups, *p* = 0.06). Conclusions: A significant proportion of AF patients received reduced doses of NOAC after ischemic stroke in a sizeable number of cases, without indication for dose reduction.

## 1. Introduction

Atrial fibrillation (AF) is the most common supraventricular arrhythmia, affecting 2–4% of adult patients [1,2]. Among individuals aged 55 years or older, the overall lifetime AF risk estimate is around 37% [3]. The most severe and dangerous AF complication is ischemic stroke (IS)—a global healthcare problem associated with high mortality, neurological impairment, and decreased quality of life. According to statistics, it is the third main cause of death and the leading cause of long-term, severe disability in people above 45 years old. In Poland, stroke is diagnosed in 80,000 individuals per year, of which 24,000 die within one year after the episode and 32,000 have a long-lasting disability [4].

AF is the cause of 15–21% of ischemic strokes [5]. In patients with AF, the ischemic stroke risk has been estimated as 1–20% annually, depending on comorbidities and history of prior cerebrovascular events [6,7]. Moreover, individuals with AF have more severe strokes compared to patients without this arrhythmia. AF has been proven to be an independent factor for early death in patients with stroke, and it is also associated with an increased risk of severe neurological deficits [4].

After an ischemic stroke, patients are at major risk of recurrent stroke. Fifty percent of them have another ischemic event within the next five years [8,9].

Oral anticoagulation therapy (OAT) is proven to be useful in reducing stroke risk [10] and it is the first-line treatment for stroke prevention in patients with AF. Currently, the most commonly used stroke risk stratification scores are CHADS_2_ and CHA_2_DS_2_-VAS_c_ [1].

Currently, the mainstay of OAT is non-vitamin K antagonist oral anticoagulants (NOACs), which are proven to be a safer option than vitamin K antagonists (VKAs) [1]. In Poland, rivaroxaban, dabigatran, and apixaban are available. According to the meta-analysis of real-world studies, these NOACs show similar associations with ischemic stroke, systemic embolism, myocardial infarction, and all-cause of death. However, apixaban is associated with lower major and gastrointestinal bleeding compared to rivaroxaban and dabigatran [11]. Each of these drugs can be used in regular or reduced doses, depending on patient characteristics. Decision to apply a reduced dose of an NOAC should be carefully considered in accordance with current guidelines.

The aim of our study is to assess the efficacy of anticoagulant therapy in patients from the POL-AF registry with a history of previous ischemic stroke.

## 2. Materials and Methods

### 2.1. Study Design and Study Population

The Polish Atrial Fibrillation (POL-AF) registry is a prospective, observational multicenter study (ClinicalTrials.gov: NCT04419012), the detailed methodology of which was described in previous publications [12,13]. It enrolled adult patients with AF from 10 cardiology hospital centers who were hospitalized from January to December 2019 (data collected from two weeks each month) and had a documented history of AF. The only exclusion criterion was AF ablation as a current reason for admittance to the hospital (according to research, these patients are usually younger and do not have concomitant diseases).

Baseline characteristics regarding demographics, medical history, type of AF, concomitant diseases, diagnostic test results, and pharmacology were collected. The estimated glomerular filtration rate (eGFR), calculated with the Chronic Kidney Disease Epidemiology Collaboration (CKD-EPI) equation, was used to assess patients’ kidney function. Thromboembolic risk and bleeding risk were defined according to the recommendations using CHA_2_DS_2_-VASc and HAS-BLED scores, respectively [14,15].

The study was approved by the Ethics Committee of the Swietokrzyska Medical Chamber in Kielce (104/2018), which also waived the requirement of obtaining informed consent from the patients.

**Figure 1:**
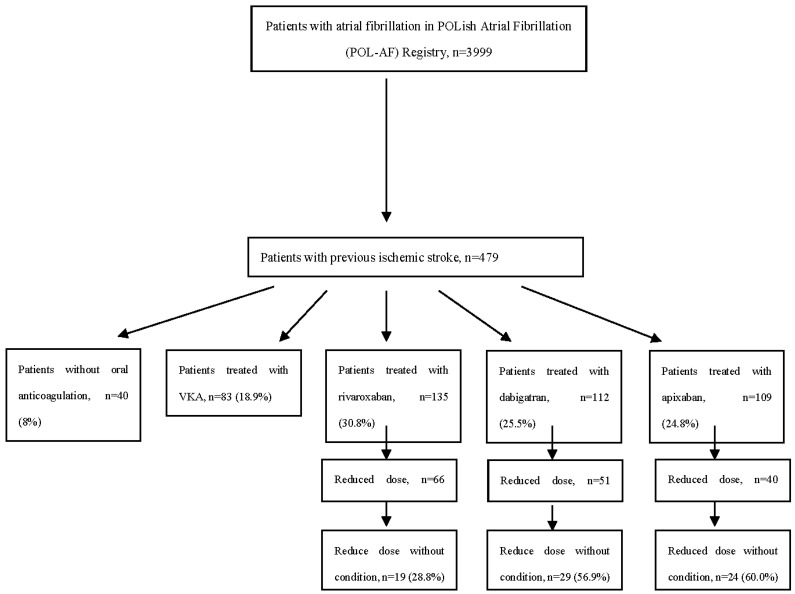
The flow chart of the study. Abbreviations: VKA, vitamin K antagonist.

In the present study, we evaluate patients from the POL-AF registry who had ischemic stroke in their medical history. The flow chart of the study is presented in Figure 1.

### 2.2. Use of Non-Vitamin K Antagonist Oral Anticoagulants (NOACs) in Stroke Prevention

An analysis of the anticoagulant therapy was performed. We especially focused on the use of reduced NOAC doses, analyzing the compliance of dose reduction with the summary of product characteristics registered with the European Medicine Agency (EMA) [16,17,18] and ESC guidelines [1]. Dose criteria were specific for each NOAC according to the following patient characteristics: weight, age, renal function, and concomitant medications. In Table 1 we present indicators for dose reduction in the study group. The univariate and multivariate analyses of factors associated with inappropriate dose reduction are presented in Table S1—Supplementary Materials.

### 2.3. Statistical Analysis

Data were collected and analyzed using MS Excel (Microsoft, 2020, version 16.40). We used the Shapiro–Wilk test to assess the distribution of variables. Data were presented as means (%) and standard deviation (SD) distributed continuous variables, medians (Me), and interquartile range (IQR) for non-normally distributed continuous variables, and as the number (N) of cases and percentage (%) for categorical variables. Statistical significance of differences between two groups was determined using the *t*-test (for comparing normal continuous variables) and the Mann–Whitney U test (for comparing non-normal continuous variables). To compare 3 groups for non-normal distributed variables we used the Kruskal–Wallis test with multiple pairwise comparisons using the Steel–Dwass–Critchlow–Fligner procedure. For categorical variables, the χ^2^ test was used. The multivariable logistic regression included all predictors with a *p* value less than 0.1. Data are presented as odds ratios (OR) with 95% confidence intervals (CI). The two-tailed *p*-value < 0.05 was considered statistically significant.

All analyses were performed using XL Stat (Addinsoft, 2020, version 2020.03.01) and MS Excel (Microsoft, 2020, version 16.40).

## 3. Results

### 3.1. Study Population

In total, 3999 patients with AF were enrolled in the POL-AF registry. Among them, 479 (12%) had previous history of IS.

Compared to patients without an IS history, post-stroke subjects were older (median age 74 years vs. 72 respectively, *p* < 0.05), 47% of them were female (42% female in group without IS history, *p* = 0.063). Permanent AF was diagnosed in 36% of subjects from the IS group (vs. 28% patients from the population without IS history, *p* < 0.001). Patients with IS had a lower body mass index in comparison to patients without a stroke history (BMI 27.7 vs. 28.7, *p* < 0.05).

Moreover, they had more comorbidities than patients without IS. In this population, there was a higher prevalence of heart failure (HF) (72% vs. 65% in group without IS history, *p* < 0.05), coronary artery disease (CAD) (63% vs. 49%, *p* < 0.001), hypertension (HT) (87% vs. 83%, *p* < 0.05), diabetes mellitus (DM) (40% vs. 33%, *p* < 0.05), peripheral artery disease (PAD) (35% vs. 12%, *p* < 0.0001), and chronic kidney disease (CKD) (32% vs. 25%, *p* < 0.05).

Furthermore, they were more likely to suffer from myocardial infarction (MI) (30% vs. 21%, *p* < 0.0001), transient ischemic attack (TIA) (8% vs. 4%, *p* < 0.0001), peripheral embolism (2% vs. 1%, *p* = 0.012), and incidents of gastrointestinal bleeding (6% vs. 4%, *p* = 0.017) in their past. Patients with an IS history have a higher CHA_2_DS_2_-VASc score compared to the group without stroke incidents (median score 7.0 vs. 4.0, *p* < 0.0001).

A comparison of the baseline characteristics, pharmacological treatment, and results of the laboratory tests of patients with and without previous ischemic stroke is presented in Table 2, Table 3 and Table 4.

### 3.2. OAC Administration

Among the 479 patients with previous IS included in this analysis, 439 (92%) had anticoagulation therapy, 83 (17%) were treated with VKA, 135 (30.8%) with rivaroxaban, 112 (25.5%) with dabigatran, and 109 (24.8%) with apixaban (Table 5).

**Figure 2:**
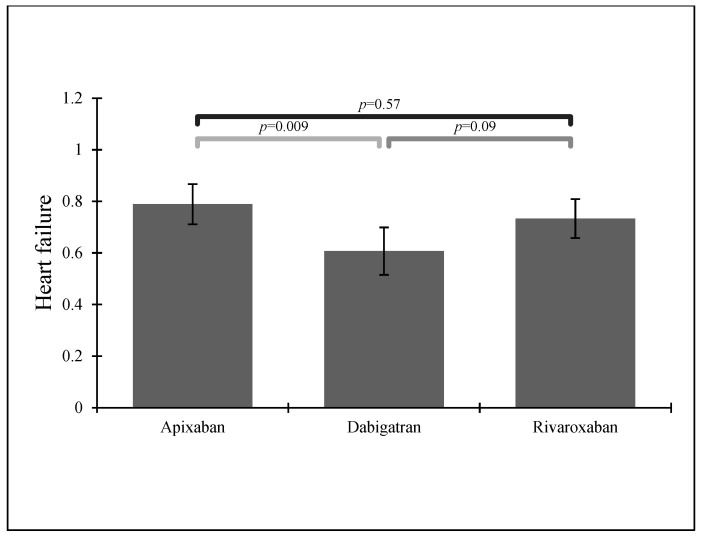
NOAC administration in patients with a previous stroke history and heart failure.

**Figure 3:**
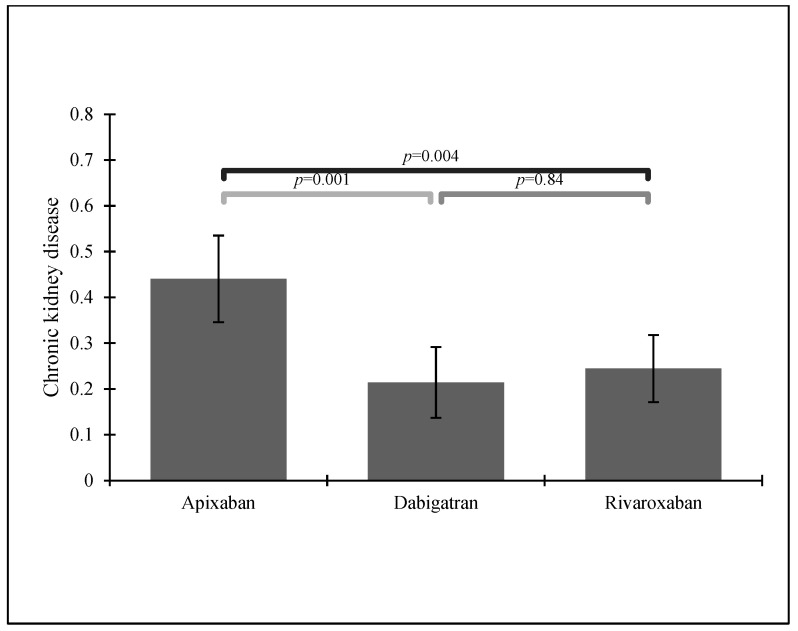
NOAC administration in patients with a previous stroke history and chronic kidney disease.

Apixaban was the most popular choice in groups of patients with HF or CKD—see Figure 2 and Figure 3.

**Figure 4:**
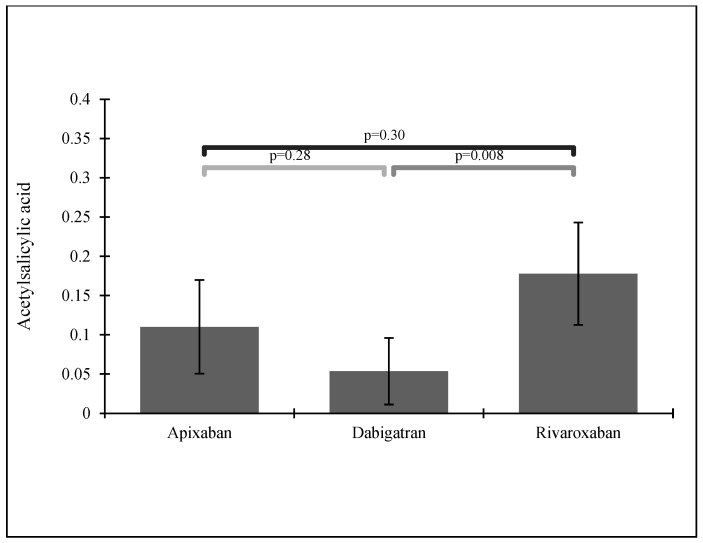
NOAC administration in patients with a previous stroke history while taking ASA.

**Figure 5:**
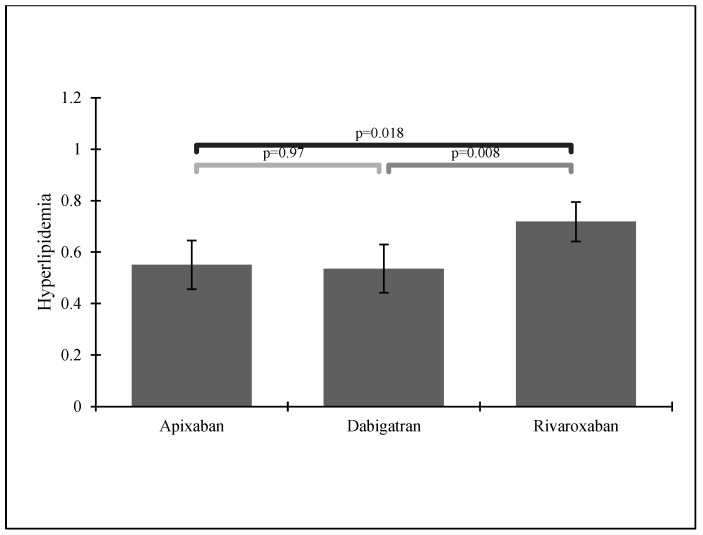
NOAC administration in patients with a previous stroke history and hyperlipidemia.

Patients taking acetylsalicylic acid (ASA) and those suffering from hyperlipidemia most often received rivaroxaban—see Figure 4 and Figure 5.

There was a significant number of patients with previous IS who were prescribed reduced doses of NOAC (48.9% for rivaroxaban, 45.5% for dabigatran, and 36.7% for apixaban). Furthermore, we established that in many cases patients were prescribed reduced doses of NOACs without indication for dose reduction (28.8% of rivaroxaban, 56.9% of dabigatran, and 60.0% of apixaban—out of groups with dose reduction, *p* = 0.06). In comparison, in the population without IS history, 32.5% of subjects received a reduced dose of rivaroxaban, 38.8% of dabigatran, and 34.8% of apixaban, and the percentages of patients with unnecessarily reduced doses were respectively 27.5%, 44.5%, and 49.0% (*p* < 0.001)—see Table 6.

## 4. Discussion

In this study, we assessed the efficacy of anticoagulant therapy in patients from the POL-AF registry with a history of previous ischemic stroke. According to our findings, these subjects are older, more often female, and they have more comorbidities in comparison to patients without a stroke history. This is consistent with previous data on this matter. Age is proven to be the most important independent risk factor of stroke development and severity and it is also a major risk factor for AF. Stroke rates double every decade after the age of 55 [19,20]. In early life, stroke is more common in men, but its incidence equalizes in the 55- to 64-year-old cohort [21]. Overall, women appear to have a higher lifetime risk of stroke and poststroke mortality, disability, depression, and dementia [19]. What is more, stroke risk related with AF also increases with age and the prevalence of AF and cardioembolic ischemic stroke is significantly higher in women than men [22].

Heart failure, hypertension, diabetes, vascular diseases, and previous incidence of thromboembolism are well-known risk factors for strokes [23], so it is not surprising that patients with a history of stroke have more comorbidities and a higher CHA_2_DS_2_-VASc score compared to the group without stroke incidence.

In patients with a history of AF, oral anticoagulation therapy remains the mainstay of stroke prevention. Although its benefits are undisputed, OAT use in especially elderly patients is often suboptimal, and in some cases is discontinued due to the risk of bleeding complications [24,25]. In our studied group, 40 subjects (8%) had no anticoagulation therapy, which is alarming considering the fact that those were patients with IS in their history. What is more, 83 (17%) patients received VKAs, which according to the guidelines is the first-choice drug only in two groups of patients: those with moderate-to-severe mitral stenosis and/or an artificial heart valve [1]. Despite the availability of three NOACs in Poland (rivaroxaban, dabigatran, and apixaban), VKAs are still commonly prescribed OACs due to their low cost. Treatment with VKAs reduces all-cause mortality by 26% and the rate of stroke by 64% [26,27], however it is proven to be a significantly less safe option than NOACs. According to meta-analysis, NOACs were associated with a significant 19% stroke/systemic embolism risk reduction, a 51% reduction in hemorrhagic stroke, and a similar ischemic stroke risk reduction compared to VKAs, but they were also associated with a significant 10% reduction in all-cause mortality and a non-significant 14% reduction in major bleeding risk [1,28]. Moreover, VKAs’ anticoagulation requires frequent control of the international normalized ratio (INR), which is often problematic. According to the research, a large proportion of patients with AF have poor VKA control, and therefore they have a higher risk of stroke or systemic embolism, major bleeding, and all-cause mortality [29]. In a previous study from our department, we observed that a history of ischemic stroke did not cause better INR control, and patients with a very high risk of thromboembolic complications more often had INR below the therapeutic range [26].

From our study, 356 patients were treated with NOACs: 135 (30.8%) with rivaroxaban, 112 (25.5%) with dabigatran, and 109 (24.8%) with apixaban. Subjects suffering from hyperlipidemia and those taking acetylsalicylic acid (ASA) most often received rivaroxaban, which was also the most popular NOAC in our group of patients. According to previous findings, rivaroxaban seems to be the most frequently used NOAC in many European countries [12,30], perhaps due to its simple dosing. Dabigatran and apixaban were equally prescribed in our group, with apixaban as the most popular choice in patients with HF or CKD.

Gold-standard randomized trials such as RE-LY with dabigatran [31], ROCKET AF with rivaroxaban [32], and ARISTOTLE with apixaban [33] had different designs regarding reduced doses. Only reduced dose of dabigatran was checked in a randomized way [31]. In the ROCKET AF trial, the dose was reduced for only 1474 patients due to chronic kidney disease [34]. In the ARISTOTLE trial only 428 patients were given a reduced dose, due to old age, low body weight, or elevated serum creatinine level [33].

In our study there was a significant number of patients with reduced doses of NOAC—48.9% for rivaroxaban, 45.5% for dabigatran, and 36.7% for apixaban. According to our research, out of groups with a reduced dose, many patients received reduced doses of NOACs without indication for dose reduction: 28.8% for rivaroxaban, 56.9% for dabigatran, and 60.0% for apixaban. Considering the fact that those subjects had experienced an ischemic stroke, this is incredibly disturbing. There is no doubt that treatment decisions for elderly patients with many comorbidities is challenging. However, as mentioned earlier, fifty percent of patients who have had an ischemic stroke have another such event within the next five years; this is the reason why OAT is crucial in these cases.

According to data from ORBIT-AF II (outcomes registry for better informed treatment of atrial fibrillation II)—a nationwide AF registry from the United States conducted from 2013 to 2016—as many as 57% of patients receiving reduced-dose NOACs did not fulfill FDA-recommended criteria for this dose. Surprisingly, they had lower bleeding risk scores than subjects receiving standard NOAC doses. They were observed to have an increased risk of adverse events, including thromboembolic events, bleeding events, and death, however they were not statistically significant [35].

In a nationwide Danish study comparing the effectiveness and safety of reduced-dose non-vitamin K antagonist oral anticoagulants and warfarin in patients with atrial fibrillation, a reduced dose of apixaban was associated with a trend toward higher rates of ischemic stroke/systemic embolism compared to warfarin, while a reduced dose of rivaroxaban and dabigatran showed a trend towards lower thromboembolic rates. However, these results were not significantly different [36].

Our data suggest that many patients are being prescribed reduced-dose NOACs, not based on the summary of product characteristics registered with the European Medicine Agency (EMA) and ESC guidelines but based on prescriber preference. As a result, this could lead to another thromboembolic event and death.

Our study has some limitations. Firstly, the POL-AF registry was an observational study designed to capture a broad selection of patients from several sites, however, selection bias cannot be excluded. Secondly, there was no long-term follow-up of patients from the POL-AF registry, therefore we cannot evaluate further prognosis in subjects after an ischemic stroke and their risk of another thromboembolic event. Thirdly, the differentiation between appropriate vs. inappropriate dosing is often dynamic and influenced by other factors not included in our study, for example the intermittent use of interacting medications.

## 5. Conclusions

A significant proportion of AF patients received reduced doses of NOACs after an ischemic stroke, in a sizeable number of cases without indication for dose reduction. As previous data suggest, those are patients requiring special clinical attention due to a markedly increased risk of another thromboembolic event. Education of physicians on the appropriate dosing of NOACs for AF patients after an ischemic stroke is required.

## Figures and Tables

**Table 1:** Detailed criteria for NOAC dose reduction in the study group.

Variables	Rivaroxaban (N = 1199)	Dabigatran (N = 819)	Apixaban (N = 953)	*p*
Dose reduction, N (%)	412 (34.3)	325 (39.7)	334 (35)	<0.001
Indication for reduction *				
Body weight ≤ 60 kg, N (%)	78 (18.9)	39 (12)	58 (17.3) **	<0.001
Age ≥ 80 years, N (%)	191 (46.4)	168 (51.7)	215 (64.4) **	<0.001
Creatinine > 1.5 mg/dL, N (%)	98 (23.8)	55 (16.9)	123 (36.8) **	<0.001
eGFR, 15–29 mL/min/1.73 m^2^, N (%)	20 (4.9)	6 (1.8)	48 (14.4)	<0.001
eGFR, 15–49 mL/min/1.73 m^2^, N (%)	194 (47.1)	95 (29.2)	80 (24)	<0.001
Dual antiplatelet therapy, N (%)	102 (24.8)	77 (23.7)	172 (51.5)	<0.001
Incidents of gastrointestinal hemorrhage, N (%)	11 (2.7)	10 (3.1)	24 (7.2)	<0.001
Incidents of cerebral hemorrhage, N (%)	6 (1.5)	4 (1.2)	12 (3.6)	<0.001
Verapamil, N (%)	1 (0.2)	1 (0.3)	4 (1.2)	<0.001
No indication for reduction, N (%)	114 (27.7)	95 (29.2)	168 (50.3)	<0.001

* numbers might not total 100% due to the fact that one patient may have had more than one indication for dose reduction. ** 2 of 3 criteria to dose reduction. Abbreviations: eGFR, estimated glomerular filtration rate; N, number.

**Table 2:** Detailed characteristics of the study group.

	Total, N = 3999	Patients without Ischemic Stroke in the Past, N = 3520	Patients with Ischemic Stroke in the Past, N = 479	*p*
Age, years	72 (16)	72 (16)	74 (15)	0.001
Female gender, N (%)	1704 (42.6%)	1481 (42%)	223 (47%)	0.06
Body mass index, kg/m^2^	28.4 (6.4)	28.7 (6.5)	27.7 (6.3)	0.005
Paroxysmal atrial fibrillation, N (%)	1923 (48.1%)	1694 (48%)	229 (48%)	0.9
Persistent atrial fibrillation, N (%)	933 (23.3%)	855 (24%)	78 (16%)	<0.001
Permanent atrial fibrillation, N (%)	1143 (28.6%)	971 (28%)	172 (36%)	<0.001
Hypertension, N (%)	3344 (83.6%)	2927 (83%)	417 (87%)	0.030
Diabetes, N (%)	1366 (34.2%)	1176 (33%)	190 (40%)	0.007
Heart failure, N (%)	2621 (65.5%)	2275 (65%)	346 (72%)	0.001
EF (%)	53% (20.0)	54% (20.0)	50% (21.3)	0.057
Coronary artery disease, N (%)	2011 (50.3%)	1710 (49%)	301 (63%)	<0.0001
Previous myocardial infarction, N (%)	894 (22.4%)	752 (21%)	142 (30%)	<0.0001
Chronic kidney disease, N (%)	1029 (25.7%)	878 (25%)	151 (32%)	0.002
Peripheral artery disease, N (%)	582 (14.6%)	414 (12%)	168 (35%)	<0.0001
Previous transient ischemic attack, N (%)	190 (4.8%)	150 (4%)	40 (8%)	<0.0001
Previous peripheral embolism, N (%)	46 (1.2%)	35 (1%)	11 (2%)	0.012
Previous incidents of gastrointestinal hemorrhage, N (%)	155 (3.9%)	127 (4%)	28 (6%)	0.017
CHA2DS2-VASc score (points)		4.0 (2)	7.0 (2)	<0.0001

Abbreviations: EF, ejection fraction; IQR, interquartile range; Me, median.

**Table 3:** Comparison of pharmacological treatment between patients with and without previous ischemic stroke.

	All N = 3999	Patients without Previous Ischemic Stroke, N = 3520	Patients with Previous Ischemic Stroke, N = 479	*p*
Acetylsalicylic acid, N (%)	607 (15.3%)	535 (15.2%)	72 (15.0%)	0.938
Clopidogrel, N (%)	539 (13.6%)	471 (13.4%)	68 (14.2%)	0.612
Ticagrelor, N (%)	5 (0.1%)	5 (0.1%)	0 (0.0%)	0.410
Diltiazem, N (%)	5 (0.1%)	4 (0.1%)	1 (0.2%)	0.580
Verapamil, N (%)	22 (0.6%)	19 (0.5%)	3 (0.6%)	0.809
Amiodarone, N (%)	751 (19.0%)	666 (18.9%)	85 (17.7%)	0.544
Beta blocker, N (%)	3398 (86.0%)	2982 (84.7%)	416 (86.8%)	0.184
Propafenone, N (%)	383 (9.7%)	345 (9.8%)	38 (7.9%)	0.195
Digoxin, N (%)	317 (8.0%)	283 (8.0%)	34 (7.1%)	0.478
Angiotensin I-converting enzyme inhibitor, N (%)	2393 (60.5%)	2103 (59.7%)	290 (60.5%)	0.713
Sartan, N (%)	722 (18.3%)	633(18.0%)	89 (18.6%)	0.741
Aldosterone receptor antagonist, N (%)	1600 (40.5%)	1394 (39.6%)	206 (43.0%)	0.146
Other diuretic, N (%)	2566 (64.9%)	2237 (63.6%)	329 (68.7%)	0.024
Dihydropyridine calcium antagonist, N (%)	1264 (32.0%)	1096 (31.1%)	168 (35.1%)	0.078
Statin, N (%)	2928 (74.1%)	2529 (71.8%)	399 (83.3%)	<0.0001

**Table 4:** Results of laboratory tests of patients with and without previous ischemic stroke.

	All N = 3999	Patients without Previous Ischemic Stroke, N = 3520	Patients with Previous Ischemic Stroke, N = 479	*p*
Hemoglobin, g/dL	13.3 (2.4)	133 (2.3)	13.1 (2.3)	0.034
White blood cells, ×10^9^/L	7.49 (2.9)	7.48 (2.9)	7.54 (3.1)	0.219
Platelet, ×10^9^/L	210 (83)	209 (83)	213 (84.5)	0.937
Creatinine, mg/dL	1.1 (0.47)	1.1 (0.5)	1.155 (0.5)	0.013
eGFR, mL/min/1.73 m^2^	60 (22.2)	60 (22)	58 (20.8)	0.004
Alanine aminotransferaze, U/L	24 (16)	24 (16)	23 (13)	0.108
Aspartate aminotransferase, U/L	26 (13)	26 (13)	27 (15)	0.853
INR	1.3 (0.6)	1.3 (0.6)	1.4 (0.6)	0.050
Uric acid, mg/dL	6.7 (2.4)	6.7 (2.3)	6.89 (2.3)	0.231
Hemoglobin A1c, %	6.45 (1.5)	6.43 (1.5)	6.6 (1.8)	0.159
Fasting glucose, mg/dL	103 (29)	103 (29)	103 (33.9)	0.756
Thyroid-stimulating hormone, uIU/mL	1.6 (1.8)	1.6 (1.8)	1.62 (1.7)	0.590
Total cholesterol, mg/dL	152 (70.3)	152 (71.3)	150.81 (63)	0.466
Low-density lipoprotein cholesterol, mg/dL	84 (64)	85 (64.7)	81.6 (60.5)	0.548
High-density lipoprotein cholesterol, mg/dL	45 (18)	45 (18)	45.62 (19.9)	0.396
Triglycerides, mg/dL	109 (70)	109.41 (69.5)	106 (72)	0.788

Abbreviations: eGFR, estimated glomerular filtration rate; INR, international normalized ratio.

**Table 5:** Anticoagulation therapy in patients with and without previous ischemic stroke.

	Nr. of Patients with Previous Ischemic Stroke (N = 479) (%)	Nr. of Patients without Previous Ischemic Stroke (N = 3520) (%)	*p*-Value for Differences between Groups	Implications for Dose Reduction in Patients with Previous Ischemic Stroke (%)	Implications for Dose Reduction in Patients without Previous Ischemic Stroke (%)	*p*-Value for Differences between Groups	Dose Reduction in Patients with Previous Ischemic Stroke (%)	Dose Reduction in Patients without Previous Ischemic Stroke (%)	*p*-Value for Differences between Groups
Oral anticoagulation therapy	439 (92%)	3172 (90%)	0.52	N/A	N/A	N/A	N/A	N/A	N/A
VKA	83 (18.9%)	557 (17.6%)	0.45	N/A	N/A	N/A	N/A	N/A	N/A
Rivaroxaban	135 (30.8%)	1064 (33.5%)	0.24	64 (47.4%)	394 (37.0%)	0.02	66 (48.9%)	346 (32.5%)	<0.001
Dabigatran	112 (25.5%)	707 (22.3%)	0.13	40 (35.7%)	231 (32.7%)	0.53	51 (45.5%)	274 (38.8%)	0.17
Apixaban	109 (24.8%)	844 (26.6%)	0.43	31 (28.4%)	259 (30.7%)	0.63	40 (36.7%)	294 (34.8%)	0.7
*p*-value for differences between NOACs	0.09	<0.001		0.007	0.01		0.17	0.03	

Abbreviations: N/A, not available; NOAC, non-vitamin K antagonist oral anticoagulant; VKA, vitamin K antagonist.

**Table 6:** Reduced doses of NOACs in patients with and without previous ischemic stroke.

	Reduction without Conditions in Patients with Previous Ischemic Stroke (%)	Reduction without Conditions in Patients without Previous Ischemic Stroke (%)	*p*-Value	Reduction with Conditions in Patients with Previous Ischemic Stroke (%)	Reduction with Conditions in Patients without Previous Ischemic Stroke (%)	*p*-Value	No Reduction with Conditions in Patients with Previous Ischemic Stroke (%)	No Reduction with Conditions without Previous Ischemic Stroke (%)	*p*-Value
Rivaroxaban	19 (28.8%)	95 (27.5%)	0.06	47 (71.2%)	251 (72.5%)	0.004	17 (12.6%)	143 (13.4%)	0.79
Dabigatran	29 (56.9%)	122 (44.5%)	0.03	22 (43.1%)	152 (55.5%)	0.66	18 (16.1%)	79 (11.2%)	0.14
Apixaban	24 (60.0%)	144 (49.0%)	0.20	16 (40.0%)	151 (51.4%)	0.18	15 (13.8%)	109 (12.9%)	0.81
*p*-value for differences between NOACs	0.06	<0.001		0.008	<0.001		0.782	0.359	

## Data Availability

Not applicable.

## Supplementary Materials

The following supporting information can be downloaded at: https://www.mdpi.com/article/10.3390/ijerph191911939/s1, Table S1: Factors associated with inappropriate dose reduction. The data are presented odds ratios with 95% confidence intervals.

## Abbreviations

AF, atrial fibrillation; ASA, acetylsalicylic acid; CAD, coronary artery disease; CKD, chronic kidney disease; DM, diabetes mellitus; HF, heart failure; HT, hypertension; INR, international normalized ratio; IQR, interquartile range; IS, ischemic stroke; Me, median; MI, myocardial infarction; NOAC, non-vitamin K antagonist oral anticoagulant; OAT, oral anticoagulation therapy; PAD, peripheral artery disease; SD, standard deviation; TIA, transient ischemic attack; VKA, vitamin K antagonist.
