# Supplementary material for: Do Patients with Atrial Fibrillation and a History of Ischemic Stroke Overuse Reduced Doses of NOACs?—Results of the Polish Atrial Fibrillation (POL-AF) Registry
Source: Int J Environ Res Public Health. 2022 Sep 21;19(19):11939. doi: 10.3390/ijerph191911939 (PMC9564626; doi:10.3390/ijerph191911939)
Supplement: Supplementary file 1 [file ijerph-19-11939-s001.zip › ijerph-1907104-supplementary.pdf]

Supplementary Materials

Table S1. Factors associated with inappropriate dose reduction. The data are presented odds ratios with 95% confidence intervals.

Variables	Apixaban (OR, 95%CI; P)		Rivaroxaban (OR, 95%CI; P)		Dabigatran (OR, 95%CI; P)	
	Univariable	Multivariable	Univariable	Multivariable	Univariable	Multivariable
Age, years	0.882 (0.781-0.996); 0.04	0.812 (0.642-1.027); 0.08	1.205 (1.055-1.377); 0.01	1.253 (0.945-1.662); 0.12	1.317 (1.155-1.502); <0.001	1.504 (1.205-1.877); <0.001
Female gender	1.134 (1.007-1.277); 0.04	1.021 (0.83-1.255); 0.85	1.044 (0.927-1.176); 0.48		1.025 (0.909-1.155); 0.69	
Weight	1.186 (1.009-1.394); 0.04	1.328 (1.046-1.688); 0.02	0.833 (0.71-0.978); 0.03	0.818 (0.627-1.068); 0.14	0.914 (0.78-1.072); 0.27	
CHA2DS2-VASc score, points	0.908 (0.805-1.023); 0.11		0.92 (0.816-1.038); 0.18		1.011 (0.896-1.14); 0.86	
Hyperlipidemia	0.859 (0.763-0.969); 0.01	0.821 (0.655-1.029); 0.09	0.88 (0.783-0.99); 0.03	1.054 (0.838-1.325); 0.65	0.888 (0.787-1.002); 0.05	1.015 (0.826-1.246); 0.89
Hypertension	1.022 (0.908-1.15); 0.72		1.058 (0.933-1.2); 0.38		0.91 (0.806-1.028); 0.13	
Diabetes mellitus	0.93 (0.826-1.047); 0.23		0.931 (0.825-1.05); 0.24		0.942 (0.835-1.063); 0.33	
Heart failure	0.981 (0.871-1.104); 0.74		0.811 (0.724-0.909); <0.001	0.976 (0.768-1.24); 0.84	0.922 (0.817-1.039); 0.18	1.232 (1.002-1.516); 0.048
EF, %	1.18 (1.026-1.357); 0.02	1.14 (1.016-1.457); 0.04	1.38 (1.178-1.616); <0.001	1.244 (0.935-1.655); 0.13	1.249 (1.08-1.443); <0.001	1.125 (0.911-1.389); 0.27
Chronic kidney disease	1.04 (0.924-1.171); 0.51		0.723 (0.631-0.829); <0.001	0.953 (0.737-1.231); 0.71	0.736 (0.645-0.839); <0.001	0.917 (0.737-1.141); 0.44
eGFR, mL/min /1.73 m2	1.187 (1.045-1.348); 0.01	1.418 (1.127-1.785); 0.003	2.343 (1.92-2.859); <0.001	2.734 (1.95-3.834); <0.001	1.675 (1.389-2.02); <0.001	3.298 (2.261-4.81); <0.001
Previous myocardial infarction	0.57 (0.442-0.736); <0.001	0.954 (0.675-1.348); 0.79	0.611 (0.437-0.856); <0.001	0.728 (0.459-1.154); 0.18	0.637 (0.502-0.809); <0.001	0.573 (0.387-0.847); 0.005
Coronary artery disease	0.652 (0.573-0.742); <0.001	0.809 (0.645-1.014); 0.07	0.72 (0.638-0.813); <0.001	0.734 (0.565-0.952); 0.02	0.68 (0.598-0.773); <0.001	0.675 (0.542-0.842); <0.001
PCI during hospitalization	0.478 (0.358-0.64); <0.001	0.014 (0.001-4.8); 0.99	0.6 (0.468-0.768); <0.001	0.557 (0.337-0.919); 0.02	0.6 (0.498-0.724); <0.001	0.563 (0.399-0.795); <0.001
Paroxysmal atrial fibrillation	0.93 (0.826-1.047); 0.23		1.088 (0.965-1.226); 0.17		0.918 (0.814-1.036); 0.16	
Persistent atrial fibrillation	1.016 (0.903-1.144); 0.79		0.979 (0.867-1.105); 0.73		1.047 (0.928-1.18); 0.46	
Permanent atrial fibrillation	1.065 (0.946-1.199); 0.3		0.927 (0.82-1.048); 0.23		1.055 (0.936-1.19); 0.38	
Peripheral artery disease	0.981 (0.871-1.104); 0.75		1.004 (0.892-1.131); 0.94		1.028 (0.912-1.16); 0.65	
Intracerebral hemorrhage	0.964 (0.85-1.092); 0.56		1.081 (0.966-1.211); 0.17		2.381 (0.071-10.855); 0.99	
Previous ischemic stroke	1.083 (0.96-1.222); 0.19		1.013 (0.901-1.14); 0.82		1.105 (0.979-1.247); 0.11	
Previous transient ischemic attack	1.028 (0.913-1.158); 0.65		0.9 (0.774-1.047); 0.17		1.124 (0.988-1.278); 0.08	
Hemoglobin, g/dL	1.03 (0.915-1.161); 0.62		1.119 (0.99-1.265); 0.07	1.089 (0.87-1.364); 0.47	1.04 (0.92-1.174); 0.53	
Previous incidents of gastrointestinal hemorrhage	1.024 (0.91-1.153); 0.69		0.998 (0.886-1.125); 0.98		1.017 (0.902-1.146); 0.79	
Dual antipatent therapy	0.011 (0.002-476.8); 0.99		0.008 (0.001-14058.6); 0.99		0.012 (0.001-961.9); 0.99	
Verapamil	0.986 (0.913-1.065); 0.72		0.963 (0.886-1.047); 0.38		1.038 (0.958-1.124); 0.36	
Left atrium diameter (mm.)	0.951 (0.883-1.024); 0.18		0.949 (0.877-1.027); 0.2		1.026 (0.933-1.129); 0.59	

Abbreviations: eGFR, estimated glomerular filtration rate; EF, ejection fraction.
